# Associations of dietary pattern, insulin resistance and risk of developing metabolic syndrome among Chinese population

**DOI:** 10.1371/journal.pone.0308090

**Published:** 2024-08-06

**Authors:** Liyong Kou, Jing Sun, Ping Wu, Zhou Cheng, Ping Zhou, Nana Li, Liang Cheng, Pengfei Xu, Yunzhuo Xue, Jiamin Tian, Wei Chen

**Affiliations:** 1 Department of General Practice, Affiliated Hospital of Jiangnan University, Wuxi, Jiangsu, People’s Republic of China; 2 Department of Nutrition, Functional Food Clinical Evaluation Center, Affiliated Hospital of Jiangnan University, Wuxi, Jiangsu, People’s Republic of China; United Arab Emirates University, UNITED ARAB EMIRATES

## Abstract

Evidence regarding the role of dietary patterns in metabolic syndrome (MetS) is limited. The mechanistic links between dietary patterns, insulin resistance, and MetS are not fully understood. This study aimed to evaluate the associations between dietary patterns and the risk of MetS in a Chinese population using a longitudinal design. Data from the China Health and Nutrition Survey, a nationally representative survey, were analyzed. MetS cases were identified based on biomarker data collected in 2009. Factor analysis was employed to identify dietary patterns, while logistic regression models were utilized to examine the association between dietary patterns and MetS. Mediation models were applied to assess multiple mediation effects. Two dietary patterns were revealed by factor analysis. Participants in the higher quartiles of the traditional Chinese dietary pattern had lower odds of MetS than those in the lowest quartile (Q1) (OR = 0.58, 95%CI: 0.48, 0.69 for Q4; OR = 0.75, 95%CI: 0.63, 0.89 for Q3). Conversely, participants in the higher quartiles of the modern Chinese dietary pattern had higher odds of MetS compared to those in the lowest quartile (Q1) (OR = 1.40, 95%CI: 1.17, 1.68 for Q4; OR = 1.27, 95%CI: 1.06, 1.52 for Q3). Significant associations between dietary patterns and MetS were mediated by insulin resistance. Therefore, dietary patterns in Chinese adults are associated with MetS, and these associations appear to be mediated through insulin resistance. These findings underscore the critical role of dietary patterns in the development of MetS and establish a foundation for culturally tailored dietary interventions aimed at reducing rates the prevalence of MetS among Chinese adults.

## 1. Introduction

Metabolic syndrome (MetS) is a clinical condition characterized by several metabolic risk factors including abdominal obesity, elevated blood pressure (BP), hyperglycemia, hypertriglyceridemia, and low high-density lipoprotein cholesterol (HDL-C) [1, 2]. In recent decades, the prevalence of MetS has increased and becoming a major public health issue all around the world [3]. In China, the prevalence of MetS has risen from 9.5% in 2002 to 18.7% in 2010–2012, with a corresponding increase in prevalence with age. This translates to an estimated 83.6 million adults living with MetS in 2002 and 189 million adults in 2010–2012 [4]. Such an increased MetS incidence extremely elevates the morbidities of arthritis, diabetes and cardiovascular disease [5, 6]. Insulin resistance is widely recognized as a central feature of MetS, although the precise mechanistic link between insulin resistance and most MetS components remains incompletely understood [7]. Therefore, it is imperative to investigate potential mechanisms of MetS and identifiy biomarkers for predicting MetS risk.

MetS arises from a complex interaction involving environmental factors, genetics, and behavior [2, 8]. The prime emphasis in the management of MetS is to mitigate the modifiable underlying risk factors through lifestyle changes [9]. Among, diet is one of the most important tools available to improve the factors linked to MetS [9]. Several studies have examined the associations between MetS and the consumption of specific food groups, individual foods, and nutrients [10–12]. Further, insulin resistance has been proposed as a postprandial phenomenon associated with acute dietary fat metabolism [13]. Several studies have demonstrated a correlation between the type of dietary fat and disruptions in insulin secretion [14, 15]. However, whether β-cell dysfunction or insulin resistance predominantly drives the association between dietary factors and MetS, particularly in Asian populations characterized by relatively lower β-cell function remains uncertain [16].

Owing to the complexity of diets and the potential associations between dietary components [17], dietary pattern analysis, which might reflect the intricacies of dietary intake [18], has emerged as an alternative and complementary approach to exploring the links between diet and risk of chronic diseases, as well as facilitating nutritional recommendations [19]. Findings from the China Health and Nutrition Survey (CHNS) illustrate an ongoing transition in dietary patterns across China [20]. Over the past two decades, dietary patterns have shifted from a traditional to less healthy options [20, 21]. This shift is characterized by an increased consumption of ultra-processed, energy-dense, and sugary foods, accompanied by a decline in the consumption of coarse grains and traditional dietary staples [20]. These profound changes in dietary patterns likely contribute to the escalating prevalence of MetS in China.

We hypothesize that different dietary patterns may impact the prevalence of MetS in the population, with insulin resistance potentially acting as a mediator in this relationship. Utilizing a well-characterized Chinese cohort, our study aims to investigate: (1) the characteristics of various dietary patterns, (2) the associations between these dietary patterns and MetS, and (3) the potential mediating role of insulin resistance in these associations.

## 2. Method

### 2.1. Study design and population

The present study used longitudinal data from CHNS, an open prospective cohort study. CHNS employed a multistage random cluster sampling process to select participants from 15 provinces across China [22]. Detailed descriptions of the methodology have been provided elsewhere [22, 23]. The study was approved by the University of North Carolina Review Board and the Ethics Review Committee of the Institute of Nutrition and Health of the Chinese Center for Disease Control and Prevention, and written informed consent was obtained from each subject at the beginning of the survey. More details are described on the survey website (http://www.cpc.unc.edu/projects/china/about/proj_desc/survey).

To assess MetS, the availability of biomarker data is essential. The biomarker data collected in the 2009 CHNS includes 26 fasting blood measures for individuals aged 7 and older. Therefore, we utilized longitudinal data from 2006 to 2009 for our analysis. We excluded participants with implausibly high or low caloric intakes (ie, < 600 or > 4000 kcal/d), those with missing dietary patterns or anthropometric data, pregnant women, and individuals with a history of metabolic disease before the baseline. This resulted in a final sample of 5918 participants from the 2006 cohort.

### 2.2. Dietary assessment and dietary patterns

Details of dietary measurements have been provided elsewhere [23]. Briefly, dietary intake was recorded for 3 consecutive days by a 24h-recall method. Foods were categorized into 20 food groups based on nutrient similarity, previous studies, and the Chinese Food Composition Table [24].

Dietary patterns were identified through factor analysis utilizing the principal component method. Factor scores were orthogonally (varimax) rotated to minimize correlation among the patterns and enhance interpretability. Dietary patterns were determined based on eigenvalues exceeding 1.5 and the scree plot. Foods with a factor loadings >|0.20| were included as the main contributors to the dietary patterns [23]. Dietary pattern scores were calculated by summing of the food factor loading coefficients and the standardized daily consumption of foods associated with each dietary pattern. These scores were then divided into quartiles, ranging from Q1 to Q4, to reflect their relative contribution to each pattern.

### 2.3. Outcome variables

Waist circumference (WC) was measured at the midpoint between the lowest rib and the iliac crest in a horizontal plane by using non-elastic tape. BP measurements were taken in triplicate by trained and qualified observers using a mercury sphygmomanometer. The mean of the three BP measurements was used in the analyses. After at least 8h of overnight fasting, blood samples were collected from household respondents aged 7 years and older. All samples were analyzed in a national central laboratory in the capital under strict quality control [8].

The homeostatic model assessment of insulin resistance (HOMA-IR) was calculated as fasting glucose (mmol/l) × fasting insulin (μU/ml)/22.5. Insulin resistance was defined as the highest quartile of HOMA-IR, as published previously [25, 26].

### 2.4. Definition of metabolic syndrome

Participants with MetS were defined according to the International Diabetes Federation criteria [27]. MetS was identified when abdominal obesity (defined as WC≥90 cm for males or ≥80 cm for females) was present along with two or more of the following criteria: 1) high blood pressure (systolic blood pressure ≥130mmHg or diastolic blood pressure≥85mmHg); 2) elevated plasma glucose (FPG≥5.6mmol/L); 3) elevated TG (TG≥1.7mmol/L); 4) low HDL-C (HDL-C<1.04mmol/L for males or <1.3mmol/L for females).

### 2.5. Covariates

Covariates were collected through interviews conducted by trained and qualified personnel, as well as through general information questionnaires. Residency was classified into two categories: urban and rural. The highest level of parental education was categorized into four levels: illiterate or primary school, middle school, high school and college or higher. The region was classified into three categories: western, eastern and central. Energy intake was calculated according to the China Food Composition and expressed in kilocalories per day (kcal/day).

### 2.6. Statistical analysis

Descriptive information (numbers and percentages) was calculated for the demographic and anthropometric characteristics of all participants. ANOVA tests for continuous variables and chi-square tests for categorical variables were used to compare the sex differences. Logistic regression models were employed to estimate the odds ratios (OR) and 95% confidence intervals (95%CI) for MetS across the quartile categories of dietary pattern scores.

Age-adjusted model was adjusted for age. Multiple-adjusted model was additionally adjusted for sex, residency, highest level of parental education, region and energy intake. Mediation models were performed using the Stata macro for multiple mediation analysis by Preacher and Hayes [28]. Different paths were produced in the model: path a represents effects of dietary pattern on mediators, path b represents effects of mediators on MetS, path c represents effects of dietary pattern on MetS not through mediators, and path a-b represents effects of dietary pattern on MetS through mediators. The bootstrapping method was applied, with coefficients estimated from 1,000 bootstrap samples [29]. A *p*-value <0.05 was regarded as statistically significant. All analyses were performed in Stata (version 13.0; StataCorp, College Sta- tion, TX, USA), and the Graphpad Prism 5.0 (GraphPad Inc., La Jolla, CA) were adopted for graph preparation.

## 3. Results

### 3.1. Factor loadings for traditional and modern dietary pattern

**Fig 1:**
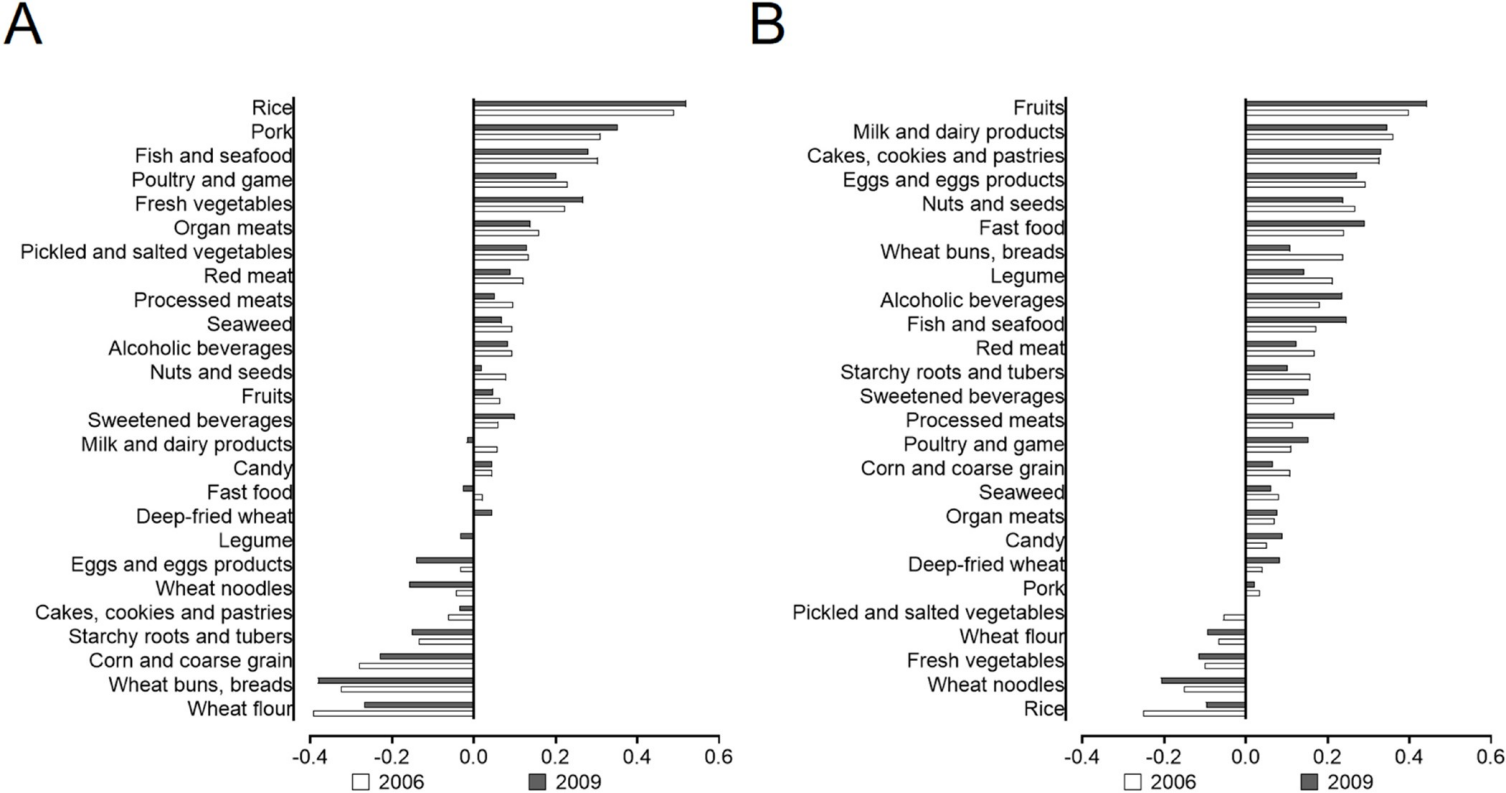
Factor loadings for traditional and modern dietary pattern. A. The Chinese dietary pattern in 2006 and 2009. B. The Modern dietary pattern in 2006 and 2009.

Two distinct dietary patterns were identified in the present study, as illustrated by factor loadings in **Fig 1**. The traditional Chinese dietary pattern exhibited high loadings on rice, vegetables, pork, fish, and seafood, while showing an inverse association with other cereals and wheat. Conversely, the modern dietary showed strong loadings on fish, fruits, milk and dairy products, cakes, cookies, pastries, with a negative loading on vegetables. Together, these two patterns accounted for 18.52% of the variance in dietary intake.

### 3.2. Characteristics of participants with and without incident MetS

**Table 1** presents the characteristics of the participants. Among the 5918 participants, 23.84% (1411/5918), were diagnosed with MetS. Participants with MetS exhibited significantly higher values of WC, BP, fasting glucose, TG, fasting insulin, and insulin resistance, along with lower levels of HDL-C (all *p*< 0.001) compared to non-cases. They were also more likely to be older, female, have lower levels of education, and reside in the eastern of China.

**Table 1:** Characteristics of participants with and without incident MetS.

Characteristic	Incident MetS		*P* value
	Non-cases (n = 4,507)	Cases (n = 1,411)	
Age (years)	52.09 (13.91)	56.79 (11.96)	<0.001
Female	2,295 (50.92)	912 (64.64)	<0.001
Residency			0.874
Urban	1,440 (31.95)	454 (32.18)	
Rural	3,067 (68.05)	957 (67.82)	
Highest level of education			<0.001
Illiterate or primary school	2,096 (46.51)	785 (55.63)	
Junior middle school	1,437 (31.88)	381 (27.00)	
High school	468 (10.38)	124 (8.79)	
College or higher	506 (11.23)	121 (8.58)	
Region			<0.001
Western	506 (11.23)	93 (6.59)	
Eastern	1,916 (42.51)	718 (50.89)	
Central	2,085 (46.26)	600 (42.52)	
Energy intake	2184.49 (630.91)	2122.11 (640.57)	0.001
Waist circumference (cm)	80.12 (9.05)	92.82 (7.67)	<0.001
Systolic blood pressure (mm Hg)	123.00 (17.80)	137.16 (19.98)	<0.001
Diastolic blood pressure (mm Hg)	79.49 (10.90)	86.76 (11.22)	<0.001
Fasting glucose (mmol/l)	5.02 (4.65, 5.45)	5.72 (5.17, 6.52)	<0.001
Triglyceride (mmol/l)	1.12 (0.79, 1.56)	2.19 (1.59, 3.06)	<0.001
HDL-C (mmol/l)	1.46 (1.25, 1.70)	1.18 (1.01, 1.38)	<0.001
Fasting insulin (mU/L)	9.47 (6.79, 13.49)	14.03 (9.59, 21.27)	<0.001
IR	793 (17.59)	683 (48.41)	<0.001

Abbreviations: HDL-C, high-density lipoprotein cholesterol; IR, insulin resistance; MetS, Metabolic syndrome.

### 3.3. Associations between dietary pattern and MetS components

The relationships between dietary patterns and MetS are displayed in **Table 2**. After adjusting for confounding factors, participants in the highest quartile of the traditional Chinese dietary pattern had lower odds of MetS compared to those in the lowest quartile (Q1) (OR = 0.58, 95%CI: 0.48, 0.69 for Q4; OR = 0.75, 95%CI: 0.63, 0.89 for Q3). Conversely, participants in the highest quartile of the modern Chinese dietary pattern had higher odds of MetS than those in the lowest quartile (Q1) (OR = 1.40, 95%CI: 1.17, 1.68 for Q4; OR = 1.27, 95%CI: 1.06, 1.52 for Q3). Further, dietary patterns showed significantly associated with most MetS components (*p*<0.05). Detailed results are presented in **Table 3**.

**Table 2:** Multivariable adjusted ORs and 95%CI for MetS according to the baseline dietary pattern scores.

	Traditional Dietary pattern					Modern Dietary Pattern				
	Q1	Q2	Q3	Q4	*P* for trend	Q1	Q2	Q3	Q4	*P* for trend
Age-adjusted Model	1	1.03 (0.87, 1.21)	0.75 (0.63, 0.89)	0.60 (0.50, 0.71)	<0.001	1	1.14 (0.95, 1.36)	1.27 (1.07, 1.51)	1.35 (1.13, 1.60)	<0.001
Multiple-adjusted model	1	1.06 (0.89, 1.25)	0.75 (0.63, 0.89)	0.58 (0.48, 0.69)	<0.001	1	1.14 (0.95, 1.36)	1.27 (1.06, 1.52)	1.40 (1.17, 1.68)	<0.001

Note: Age-adjusted model: adjusted for age. Multiple-adjusted model: additional adjusted for sex, residency, highest level of parental education, region and energy intake. *p*-value for trend was obtained by adjusting the quartile of the pattern scores as a continuous variable. *p*-values<0.05 are bold. Abbreviations: MetS, Metabolic syndrome.

**Table 3:** Associations between dietary pattern and MetS components.

	Traditional Dietary pattern					Modern Dietary Pattern				
	Q1	Q2	Q3	Q4	*P* for trend	Q1	Q2	Q3	Q4	*P* for trend
Abdominal obesity	1	0.80 (0.69, 0.94)	0.53 (0.46, 0.62)	0.48 (0.41, 0.56)	<0.001	1	1.33 (1.14, 1.55)	1.26 (1.08, 1.47)	1.49 (1.27, 1.75)	<0.001
High blood pressure	1	1.01 (0.86, 1.18)	0.82 (0.71, 0.96)	0.89 (0.80, 1.05)	0.034	1	1.15 (0.98, 1.35)	1.29 (1.10, 1.51)	1.27 (1.08, 1.50)	0.001
Elevated plasma glucose	1	0.84 (0.71, 0.99)	0.90 (0.77, 1.06)	0.77 (0.65, 0.92)	0.010	1	1.06 (0.90, 1.25)	0.95 (0.80, 1.13)	1.05 (0.88, 1.24)	0.924
Elevated TG	1	1.23 (1.05, 1.44)	1.08 (0.93, 1.27)	1.02 (0.86, 1.19)	0.908	1	0.99 (0.85, 1.17)	1.20 (1.03, 1.41)	1.22 (1.04, 1.44)	0.002
Low HDL-C	1	1.07 (0.90, 1.26)	1.00 (0.85, 1.18)	0.83 (0.70, 0.99)	0.054	1	1.09 (0.91, 1.29)	1.31 (1.11, 1.56)	1.17 (0.98, 1.40)	0.017

Note: Multiple-adjusted model: adjusted for age, sex, residency, highest level of parental education, region and energy intake. *p*-value for trend was obtained by adjusting the quartile of the pattern scores as a continuous variable. *p*-values<0.05 are bold. Abbreviations: HDL-C, high-density lipoprotein cholesterol; MetS, Metabolic syndrome; TG, triglyceridemia.

### 3.4. Multiple mediation models of IR in the associations between dietary pattern and MetS

After adjusting for confounding variables, the traditional dietary pattern showed an inverse association with insulin resistance, whereas the modern dietary pattern exhibited a positive association with insulin resistance (*p*<0.05). Further multiple mediation analysis substantiated that the significant associations between dietary patterns and MetS were partially mediated by IR (mediation proportion: 20.31% for traditional dietary pattern; 17.96% for modern dietary pattern). Detailed multiple mediation effects are shown in **Table 4**.

**Table 4:** Multiple mediation models of insulin resistance in the associations between dietary pattern and MetS.

	DP on mediator	*P* value	Direct effect	*P* value	Indirect effect	*P* value	Proportion mediated (%)
	(X M)		(X Y, adjusted M)		(X M Y)		
Insulin resistance							
Traditional DP	-0.0228 (-0.0330, -0.0126)	<0.001	-0.0255 (-0.0349, -0.0160)	<0.001	-0.0065 (-0.0095, -0.0036)	<0.001	20.31
Modern DP	0.0099 (0.0011, 0.0198)	0.049	0.0137 (0.0045, 0.0230)	0.004	0.0030 (0.0001, 0.0059)	0.049	17.96

Note: Multiple-adjusted model: adjusted for age, sex, residency, highest level of parental education, region and energy intake. *p*-values<0.05 are bold. Abbreviations: MetS, Metabolic syndrome; Modern DP, Modern Dietary Pattern; Traditional DP, Traditional Dietary pattern.

## 4. Discussion

As previously mentioned, we identified two major dietary patterns in the present study: the traditional Chinese dietary pattern and the modern Chinese dietary pattern. These patterns effectively captured eating habits, explaining 18.52% of the variance in dietary intake. The identified “traditional” and “modern” Chinese dietary patterns align with previous findings in China [24, 30, 31]. Our results indicate that adherence to the traditional dietary pattern was associated with a decreased risk of MetS, while adherence to the modern dietary pattern was associated with an increased risk. Besides, the association between dietary patterns and MetS was mediated by insulin resistance.

The traditional dietary pattern identified in the present study was associated with a decreased risk of MetS, consistent with previous studies [32, 33], indicating that healthy dietary patterns are inversely associated with MetS. This inverse association may be attributed to the pattern’s rich composition of vitamins, minerals, fiber, and omega-3 fatty acids, which are known for their protective effects against MetS and its components [34]. For instance, a meta-analysis of nine studies demonstrated that higher vegetable consumption is associated with a lower risk of MetS [35]. Additionally, diets rich in whole grains and vegetables are associated with increased dietary fiber intake, particularly soluble fiber, which is linked to a reduced risk of MetS [36]. Furthermore, despite ongoing debate, rice consumption, primarily within Asiatic dietary patterns, may not increase the risk of developing MetS. Rice is a low-energy food with lower energy density compared to wheat [37], attributed partly to differences in cooking methods. Steamed rice, for example, contains twice the amount of water and half the energy content per equivalent serving of bread [38]. Moreover, traditional dietary patterns often include a high intake of white meat, fish, and seafood. White meat is lower in fat compared to red meat, with primarily beneficial unsaturated fats [39]. Fish, particularly rich in omega-3 polyunsaturated fatty acids, has been linked to improvements in MetS by reducing plasma triglyceride levels [40]. Supplementation with omega-3 polyunsaturated fatty acids has shown potential in improving metabolic syndrome features such as insulin resistance, hypertension, and dyslipidemia by decreasing plasma triglycerides [40].

The present study also identified an energy-dense, high-fat, and sugary dietary pattern associated with an increased risk of MetS. This finding aligns with previous research indicating a positive association between the Western dietary pattern and MetS [32, 41]. Several explanations may account for this association. Firstly, modern dietary patterns characterized by high consumption of animal products such as meat, eggs, and dairy, which are rich in saturated fat and cholesterol, have been linked to increased risks of obesity, hypertension, and dyslipidemia [39, 42]. Secondly, higher consumption of refined grains has been associated with elevated odds of MetS [12]. This can be attributed to the varying glycemic indices of different refined-grain foods. High glycemic index foods lead to higher postprandial blood glucose levels compared to those with a low glycemic index, thus increasing long-term insulin demand [43]. Thirdly, dietary sugars contribute excess energy and rapidly absorbable carbohydrates, which are implicated in the development of MetS [10]. Additionally, the consumption of fast food has also been linked to MetS due to its high content of saturated and trans-saturated fats, as well as salt [44].

The median of HOMA-IR in our study was 2.34 (interquartile range, 1.60–3.66), consistent with values previously reported in China [45]. Insulin resistance was defined as the highest quartile of HOMA-IR. Interestingly, we observed that the association between dietary patterns and MetS appeared to be mediated by insulin resistance, particularly in the traditional Chinese dietary pattern. Several explanations may account for this association. Firstly, vegetables are rich in vitamins, minerals, and phytochemicals, which can enhance insulin production, improve insulin sensitivity, and potentially prevent MetS [22, 26, 35]. Secondly, meat consumption provides substantial amounts of saturated fatty acids, notably palmitic acid [39, 46], which may inhibit insulin receptor substrate-1, phosphatidylinositol-3-kinase, or protein kinase B in adipocytes, contributing to insulin resistance [47]. Thirdly, dietary fiber intake can influence metabolism by slowing carbohydrate absorption and digestion, thereby reducing the insulin demand [48].

However, it is important to acknowledge several limitations. Our study was constrained by the use of older data sources, and we attempted to select 2006 baseline data to compare with 2009 outcomes to better assess the impact of dietary patterns on metabolic syndrome. Unfortunately, due to database limitations, metabolic indicators for the cohort population were not available for the 2006 baseline, limiting our ability to examine the effect of early dietary patterns on advanced metabolic syndrome outcomes in 2009. And, dietary data were based on baseline assessments, which may not fully capture long-term dietary habits. Although factor loadings for dietary patterns were similar between 2006 and 2009, this should be considered. In addition, subjective decisions such as the number of factors retained and the labeling of dietary patterns were made, which are inherent to factor analyses; however, our methodology has been validated and deemed satisfactory [22]. Finally, despite controlling for numerous covariates, potential confounding factors related to socioeconomic status and genetics may have influenced our findings.

## 5. Conclusion

In conclusion, this study identified two distinct dietary patterns among Chinese adults. The traditional Chinese dietary pattern was inversely associated with MetS, whereas the modern dietary pattern showed a positive association. These associations were mediated through insulin resistance in Chinese adults, highlighting the significant role of dietary patterns in preventing and managing MetS. Certainly, further research is essential to validate these findings, explore their determinants, and assess their potential applicability in diverse populations.

## Supporting information

S1 Graphical abstract This study evaluated the relationship between dietary patterns and the risk of metabolic syndrome (MetS) in a population through a longitudinal analysis of data from the Chinese Health and Nutrition Survey. A significant association between dietary patterns and MetS was identified. Specifically, modern dietary patterns in Chinese adults were positively correlated with an increased risk of MetS, with insulin resistance mediating these associations. Conversely, traditional dietary patterns were inversely associated with MetS.
(TIF)

## Abbreviations

BP: Blood pressure
CHNS: China Health and Nutrition Survey
HDL-C: High-density lipoprotein cholesterol
HOMA-IR: Homeostatic model assessment of insulin resistance
MetS: Metabolic syndrome
Modern DP: Modern Dietary Pattern
WC: Waist circumference
OR: odds ratios
TG: Triglyceridemia
Traditional DP: Traditional Dietary pattern
95% CI: 95% confidence intervals
