# Supplementary figures and images for: Associations of dietary pattern, insulin resistance and risk of developing metabolic syndrome among Chinese population
Source: PLoS One. 2024 Aug 6;19(8):e0308090. doi: 10.1371/journal.pone.0308090 (PMC11302861; doi:10.1371/journal.pone.0308090)

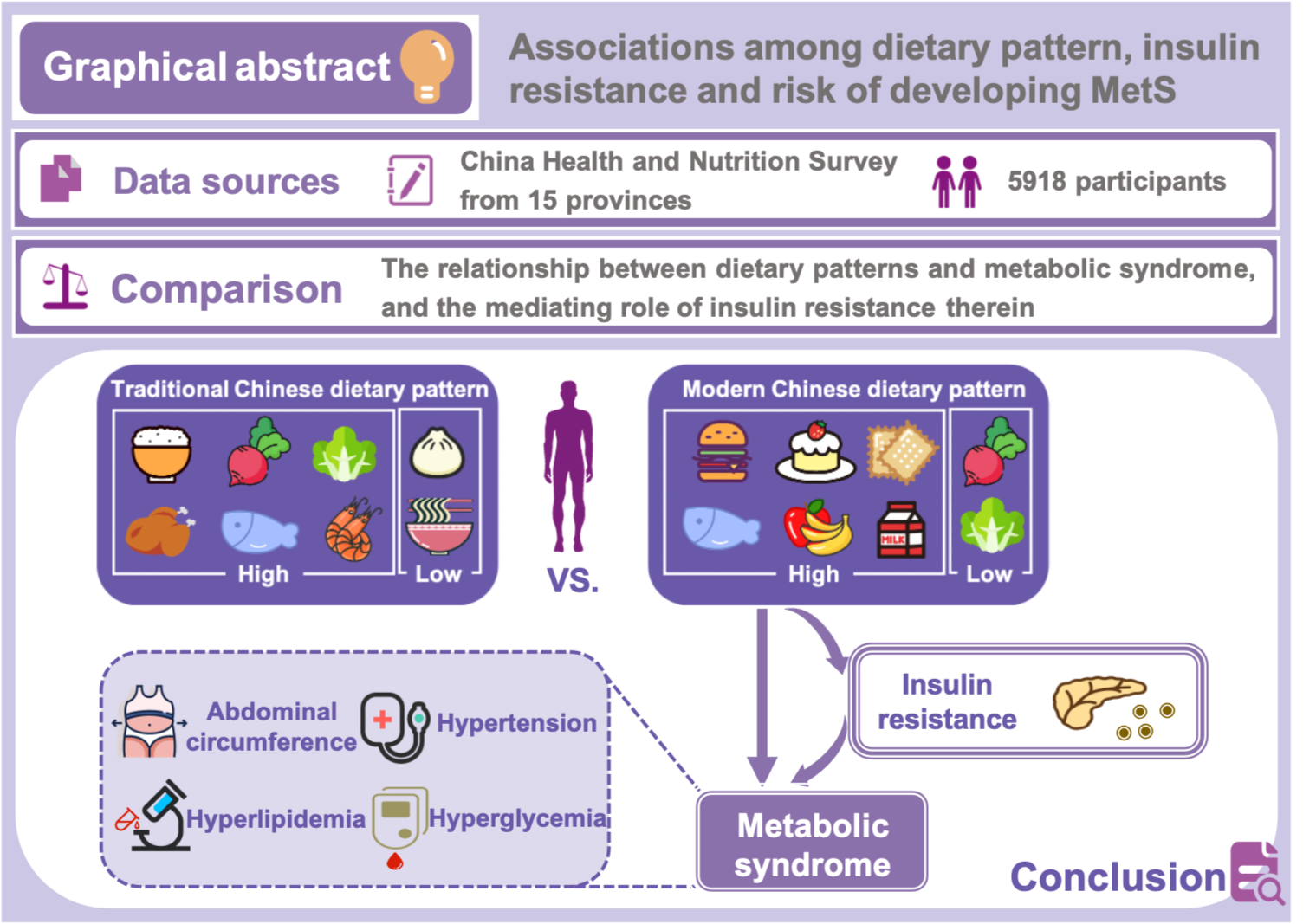

Supplement: S1 Graphical abstract — A significant association between dietary patterns and MetS was identified. Specifically, modern dietary patterns in Chinese adults were positively correlated with an increased risk of MetS, with insulin resistance mediating these associations. Conversely, traditional dietary patterns were inversely associated with MetS. (TIF) [file pone.0308090.s001.tif]
